# Aesculetin Inhibits Osteoclastic Bone Resorption through Blocking Ruffled Border Formation and Lysosomal Trafficking

**DOI:** 10.3390/ijms21228581

**Published:** 2020-11-13

**Authors:** Woojin Na, Eun-Jung Lee, Min-Kyung Kang, Yun-Ho Kim, Dong Yeon Kim, Hyeongjoo Oh, Soo-Il Kim, Su Yeon Oh, Young-Hee Kang

**Affiliations:** Department of Food Science and Nutrition and The Korean Institute of Nutrition, Hallym University, Chuncheon 24252, Korea; nsm0729@hanmail.net (W.N.); reydmswjd@naver.com (E.-J.L.); mitholy@hallym.ac.kr (M.-K.K.); royalskim@hallym.ac.kr (Y.-H.K.); ehddus3290@naver.com (D.Y.K.); ohhyeongju@gmail.com (H.O.); ky4850@naver.com (S.-I.K.); suy0411@naver.com (S.Y.O.)

**Keywords:** aesculetin, bone resorption, lysosomes, microtubules, osteoclast, ruffled border

## Abstract

For the optimal resorption of mineralized bone matrix, osteoclasts require the generation of the ruffled border and acidic resorption lacuna through lysosomal trafficking and exocytosis. Coumarin-type aesculetin is a naturally occurring compound with anti-inflammatory and antibacterial effects. However, the direct effects of aesculetin on osteoclastogenesis remain to be elucidated. This study found that aesculetin inhibited osteoclast activation and bone resorption through blocking formation and exocytosis of lysosomes. Raw 264.7 cells were differentiated in the presence of 50 ng/mL receptor activator of nuclear factor-κB ligand (RANKL) and treated with 1–10 μM aesculetin. Differentiation, bone resorption, and lysosome biogenesis of osteoclasts were determined by tartrate-resistance acid phosphatase (TRAP) staining, bone resorption assay, Western blotting, immunocytochemical analysis, and LysoTracker staining. Aesculetin inhibited RANKL-induced formation of multinucleated osteoclasts with a reduction of TRAP activity. Micromolar aesculetin deterred the actin ring formation through inhibition of induction of αvβ3 integrin and Cdc42 but not cluster of differentiation 44 (CD44) in RANKL-exposed osteoclasts. Administering aesculetin to RANKL-exposed osteoclasts attenuated the induction of autophagy-related proteins, microtubule-associated protein light chain 3, and small GTPase Rab7, hampering the lysosomal trafficking onto ruffled border crucial for bone resorption. In addition, aesculetin curtailed cellular induction of Pleckstrin homology domain-containing protein family member 1 and lissencephaly-1 involved in lysosome positioning to microtubules involved in the lysosomal transport within mature osteoclasts. These results demonstrate that aesculetin retarded osteoclast differentiation and impaired lysosomal trafficking and exocytosis for the formation of the putative ruffled border. Therefore, aesculetin may be a potential osteoprotective agent targeting RANKL-induced osteoclastic born resorption for medicinal use.

## 1. Introduction

Old and damaged bones are replaced with newly formed ones during a highly controlled bone remodeling process that is crucial for preserving skeleton integrity and mineral homeostasis [1,2]. Normal bone remodeling entails a tight coupling of osteoclastic bone resorption to osteoblastic bone formation, maintaining optimal bone mass or quality [2,3]. However, this physiological process can be deranged by various factors such as menopause-associated hormonal changes, age-related factors, changes in physical activity, drugs, and secondary diseases [2]. Numerous studies have elucidated the cellular and molecular mechanisms that regulate the finely coordinated bone remodeling involving activation, resorption, reversal, formation, and termination [4,5]. The key signaling pathways regulating osteoclastogenesis and osteoblastogenesis are known to be receptor activator of nuclear factor-κB (RANK)/RANK ligand (RANKL)/osteoprotegerin (OPG) and canonical Wnt signaling [6,7,8]. Additionally, bone remodeling involves several paracrine and endocrine regulators such as cytokines and growth factors [1,9]. Understanding diverse mechanisms underlying bone remodeling provides targets for pharmacological interventions [10,11,12]. Currently available osteoanabolic therapies are the parathyroid hormone and its related peptide synthetic analogues, and antiresorptive therapies include bisphosphonates or denosumab [10,12]. However, definite data are needed to confirm the efficacy and safety of such therapeutic interventions.

Osteoclasts are multinucleated hematopoietic cells that are specialized for bone resorption [7,8]. Emerging findings reveal evidence that the ruffled border of mature osteoclasts in contact with the bone surface has discrete subdomains that are operated by several membrane trafficking pathways in osteoclasts [13]. The ruffled border is formed by massive fusion of secretory lysosomes filled with resorptive enzymes [14,15]. Bone resorption by osteoclasts highly depends on proteases, acid hydrolases and acid phosphatases including tartrate resistant acid phosphatase (TRAP), matrix metalloproteinases (MMP), and cathepsin K [13]. Additionally, the bone surface acidification is responsible for digesting inorganic materials of bone matrices [15,16]. Osteoclastic function of resorbing the mineralized bone matrix depends on formation of resorption lacuna presenting specific proteases and low pH [13]. Defect of proteases such as TRAP, MMP, and cathepsin K fails to digest organic components of bone matrices [17]. The inactivation of ruffled border proteins essential for the extracellular acidification results in osteopetrosis manifested by dense bones [17,18,19].

The molecular machinery governing positioning and peripheral distribution of lysosomes has recently emerged [13,20]. Several intersecting endocytic, secretory, transcytotic, and autophagic pathways take place during the formation discrete subdomains of the ruffled border. Lysosome trafficking depends on regulation of cytoskeletal and lysosomal proteins of Pleckstrin homology domain-containing protein family member 1 (PLEKHM1) and lissencephaly-1 (LIS1) [21,22]. The lysosomal membrane proteins of synaptotagmin VII (Syt VII) and lysosome-associated membrane protein 2 (LAMP2) regulate cathepsin K secretion and formation of the ruffled border in osteoclasts. Loss of connectivity of lysosomes to microtubules impairs bone resorption and lysosomal distribution in osteoclasts [20]. In addition, formation of the ruffled border entails several autophagy-related proteins (Atg) in concert with microtubule-associated protein light chain 3 (LC3) for actin ring formation, cathepsin K release, and bone resorption of osteoclasts [23,24]. However, the molecular mechanisms regulating lysosomal biogenesis and function in osteoclasts remain unclear. Distinct vesicle transport and protrusion along microtubules in osteoclasts may be exploited for the development of new therapies for metabolic bone disorders.

**Figure 1:**
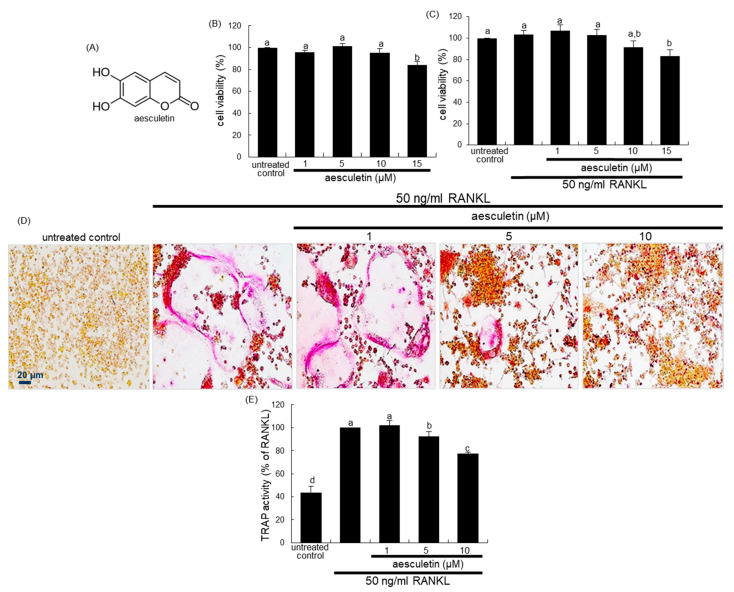
Chemical structure of aesculetin (**A**), cytotoxicity of Raw 264.7 macrophages by 1–15 µM aesculin in the absence (**B**) and presence of receptor activator of nuclear factor-κB ligand (RANKL) (**C**), and inhibitory effects of aesculetin on tartrate-resistance acid phosphatase (TRAP) staining (**D**) and TRAP activity (**E**). Raw 264.7 cells were cultured for 3 days with 1–15 µM aesculetin in the absence and presence of 50 ng/mL RANKL. Cell viability was measured by 3-(4,5-dimetylthiazol-yl)-diphenyl tetrazolium bromide (MTT) assay (**B**,**C**). Bar graphs for viability (mean ± SEM, *n* = 3) are expressed as percentage cell survival compared to untreated cells. For the TRAP staining and activity (**D**,**E**), Raw 264.7 cells were cultured for 5 days with 50 ng/mL RANKL in the absence or presence of 1–10 µM aesculetin. TRAP-positive multinucleated osteoclasts were observed under light microscopy (four separate experiments). Scale bar = 20 µm. TRAP activity (mean ± SEM, *n* = 3) was measured at λ = 405 nm. Respective values in bar graphs not sharing a small letter are significantly different at *p* < 0.05.

**Figure 2:**
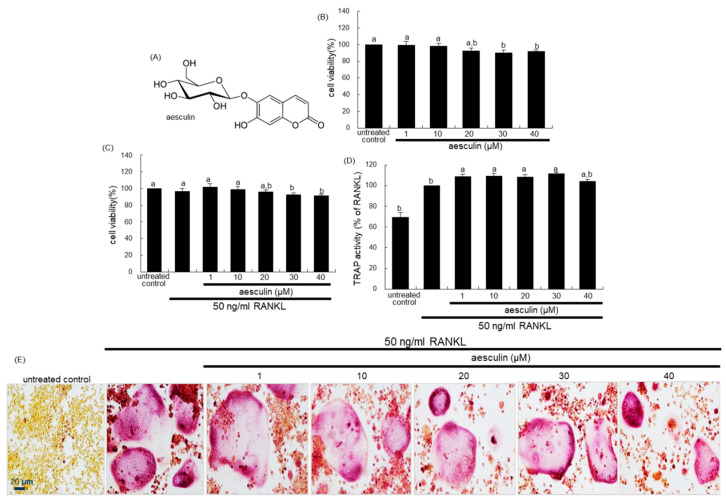
Chemical structure of aesculin (**A**), cytotoxicity of Raw 264.7 macrophages by 1–40 µM aesculin (**B**,**C**), and inhibitory effects of aesculin on tartrate-resistance acid phosphatase (TRAP) activity (**D**) and staining (**E**). Raw 264.7 cells were cultured for 3 days with 1–40 µM aesculin in the absence and presence of 50 ng/mL receptor activator of nuclear factor-κB ligand (RANKL). Cell viability was measured by 3-(4,5-dimetylthiazol-yl)-diphenyl tetrazolium bromide (MTT) assay (**B**,**C**). Bar graphs for viability (mean ± SEM, *n* = 3) are expressed as percentage cell survival compared to untreated cells. For the TRAP activity and staining (**D**,**E**), Raw 264.7 cells were cultured for 5 days with 50 ng/mL RANKL in the absence or presence of 1–40 µM aesculin. TRAP activity (mean ± SEM, *n* = 3) was measured at λ = 405 nm. TRAP-positive multinucleated osteoclasts were observed under light microscopy (four separate experiments). Scale bar = 20 µm. Respective values in bar graphs not sharing a small letter are significantly different at *p* < 0.05.

There have been considerable efforts in developing novel therapeutic agents targeting bone loss with natural plant-derived compounds that do not exhibit any undesirable effects [25,26]. However, the mechanistic efficacy of these compounds in counteracting bone loss remains elusive. Gossypetin, a bioactive compound of *Hibiscus sabdariffa*, antagonized bone resorption through inhibiting the ruffled border formation [27]. On the basis of the evidence that bone resorption in osteoclasts relies on a molecular apparatus linking lysosomes to microtubules, the present study examined whether aesculetin (Figure 1A) and its glucoside aesculin (Figure 2A) inhibited bone resorption through disturbing the lysosomal intracellular trafficking to the ruffled border in RANKL-exposed murine Raw 264.7 macrophages.

## 2. Results

### 2.1. Blockade of RANKL-Induced Osteoclast Differentiation by Aesculetin

Mature osteoclasts are multinucleated giant cells differentiated by the RANKL/RANK system from their precursors [6,7]. To determine the inhibitory effect of nontoxic aesculetin on the osteoclast differentiation, Raw 264.7 macrophages were treated with 1–10 μM aesculetin in the presence of 50 ng/mL RANKL. RANKL promoted the differentiation of Raw 264.7 macrophages into TRAP-positive multinucleated osteoclasts (Figure 1D,E). However, there was a marked inhibition of the formation of TRAP-positive osteoclasts in the presence of ≥5 µM aesculetin (Figure 1D). Consistently, the TRAP activity enhanced by RANKL was reduced in ≥ 5 µM aesculetin-added cells (Figure 1E). On the other hand, 1–40 µM aesculin did not reduce the TRAP activity significantly in RANKL-loaded Raw 264.7 cells (Figure 2D). In addition, nontoxic aesculin did not influence the formation of TRAP-positive multinucleated osteoclasts by RANKL (Figure 2E). Accordingly, aesculetin but not aesculin within the limits of cytotoxicity may inhibit the differentiation of osteoclasts from common progenitor cells including macrophages.

### 2.2. Inhibition of Lacunar Acidification and Bone Resorption by Aesculetin

**Figure 3:**
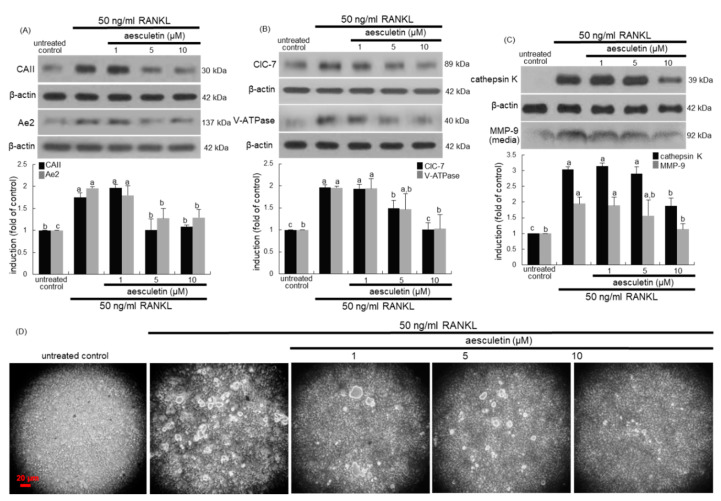
Inhibition of induction of carbonic anhydrase II (CAII), anion exchange protein 2 (Ae2), chloride channel 7 (ClC-7), vacuolar-type H(+)-ATPase (V-ATPase), and cathapsin K (**A**,**B**), matrix metalloproteinase 9 (MMP-9) secretion (**C**), and bone resorption (**D**) by aesculetin. Raw 264.7 cells were cultured in minimum essential medium alpha medium (α-MEM) with 50 ng/mL receptor activator of nuclear factor-κB ligand (RANKL) in the absence or presence of 1–10 μM aesculetin for 5 days. Whole-cell lysates were subject to SDS-PAGE and Western blot with a specific antibody against CAII, Ae2, ClC-7 V-ATPase, and cathapsin K (**A**,**B**). An equal volume of culture medium was subject to sodium dodecyl sulphate-polyacrylamide gel electrophoresis (SDS-PAGE) and Western blot with a specific antibody against MMP-9 (**C**). β-Actin was used as internal control. The bar graphs (mean ± SEM, *n* = 3) represent quantitative results of blots obtained from a densitometer. Respective values in double-bar graphs not sharing a small letter are significantly different at *p* < 0.05. The osteoclast bone resorption was assayed using a commercially available bone resorption assay kit ((**D**), three separate experiments). Attached cells were removed, and resorption pits on the plate were visualized under light microscopy. Scale bar = 20 µm.

The carbonic anhydrase II (CAII) offers the proton source for extracellular acidification, and the Cl^−^/HCO3^−^ exchanger anion exchange protein 2 (Ae2) can serve as cellular entry mechanism for Cl^−^ during bone resorption [16,18]. RANKL promoted CAII induction, while ≥ 5μM aesculetin attenuated any such induction (Figure 3A). In addition, the RANKL induction of Ae2 protein was suppressed by administering ≥ 5 µM aesculetin to Raw 264.7 macrophages (Figure 3A). On the other hand, the chloride channel 7 (ClC-7) and the proton-pumping vacuolar-type H(+)-ATPase (V-ATPase) localized to the ruffled membrane contribute to lacunar acidification of the osteoclast-bone interface [16,19]. Similarly, aesculetin reduced the cellular levels of ClC-7 and V-ATPase enhanced in the presence of 50 ng/mL RANKL (Figure 3B), indicating that aesculetin deterred the acidification of resorptive microenvironment in osteoclasts.

Osteoclasts secrete lytic enzymes of proteases and acid hydrolases, as well as TRAP-degrading calcified bone matrix in the resorption lacunae [6,7]. Cathepsin K is a lysosomal cysteine protease which plays an essential role in bone resorption [28]. RANKL enhanced the cellular expression of cathepsin K in Raw 264.7 macrophages, which was markedly attenuated by administering 10 µM aesculetin (Figure 3C). In addition, the secretion of MMP-9 was upregulated by adding RANKL to macrophages (Figure 3C). However, the administration of 10 µM aesculetin to RANKL-loaded macrophages reduced the secretion of MMP-9. In fact, MMP-9 is highly induced in osteoclasts by RANKL signals and degrades bone collagens in concert with cathepsin K in the osteoclastic microenvironment [29].

To examine the effect of aesculetin on the bone-resorbing activity of osteoclasts, Raw 264.7 cells were cultured on calcium phosphate-coated plates in the presence of 50 ng/mL RANKL and treated with 1–10 μM aesculetin. The osteoclasts created several resorption pits, but aesculetin was effective in diminishing the pit areas (Figure 3D). Collectively, aesculetin may retard bone resorption through hampering serine proteases and MMP including cathepsin K and MMP-9.

### 2.3. Inhibitory Effects of Aesculetin on Actin Ring Formation

**Figure 4:**
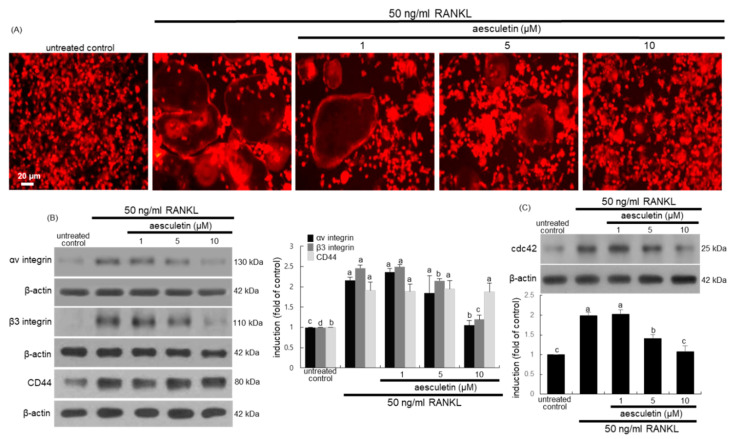
Blockade of receptor activator of nuclear factor-κB ligand (RANKL)-induced actin ring formation (**A**) and induction of integrins, cluster of differentiation 44 (CD44) and Cdc42 (**B**,**C**) in mature osteoclasts. Raw 264.7 cells were cultured for 5 days with 50 ng/mL RANKL in the absence or presence of 1–10 µM aesculetin. Differentiated cells were fixed in 4% formaldehyde for 10 min, and fluorescent rhodamine phalloidin was added to fixed cells and hence incubated for 20 min at 4 °C (**A**). Fluorescent images were taken with an Axiomager Optical fluorescence microscope. Original magnification of microscopic images (*n* = 3). Scale bar = 20 µm. After whole-cell extracts were subject to sodium dodecyl sulphate-polyacrylamide gel electrophoresis (SDS-PAGE) and Western blot analysis with a primary antibody against αv integrin, β3 integrin, CD44, and Cdc42 (**B**,**C**). Representative blot data were obtained from three independent experiments, and β-actin protein was used as an internal control. The bar graphs (mean ± SEM) represent quantitative results obtained from a densitometer. Respective values in different-colored bar graphs not sharing a small letter indicate a significant difference at *p* < 0.05.

The actin ring formation of osteoclasts is a critical prerequisite for optimal osteoclastic resorption [30]. The formation of actin rings was elevated in RANKL-treated macrophages, as evidenced by red staining of rhodamine phalloidin (Figure 4A). When ≥ 5 μM aesculetin was administered to RANKL-loaded macrophages, the formation of actin rings was highly attenuated.

The αvβ3 integrin abundantly expressed in osteoclasts is localized in the sealing zone, known as a specialized cell–matrix adhesion structure [31,32]. This study determined whether aesculetin suppressed the induction of αvβ3 integrin at the podosome cloud of RANKL-exposed macrophages. The induction of αv integrin and β3 integrin was markedly promoted by administering 50 ng/mL RANKL to Raw 264.7 cells (Figure 4B). In contrast, ≥5 μM aesculetin markedly inhibited the induction of both integrin proteins. On the other hand, the cluster of differentiation 44 (CD44) is localized in the sealing zone of osteoclasts and involved in actin cytoskeletal organization by linking to podosome cores [32,33]. RANKL enhanced the induction of core-linking CD44, but aesculetin did not influence the cellular CD44 induction of osteoclasts (Figure 4B). Consequently, aesculetin may suppress formation of the sealing zone required for effective osteoclastic bone resorption through inhibiting the induction of diffuse cloud-associated αvβ3 integrin. Furthermore, the small GTPase Cdc42 regulates assembly and organization of podosomes into the sealing zone of osteoclasts [34]. The cellular Cdc42 induction was highly enhanced in RANKL-loaded Raw 264.7 cells, while ≥ 5 μM aesculetin attenuated its induction (Figure 4C).

### 2.4. Disruption of Osteoclastic Cytoskeletal Arrangement by Aesculetin

**Figure 5:**
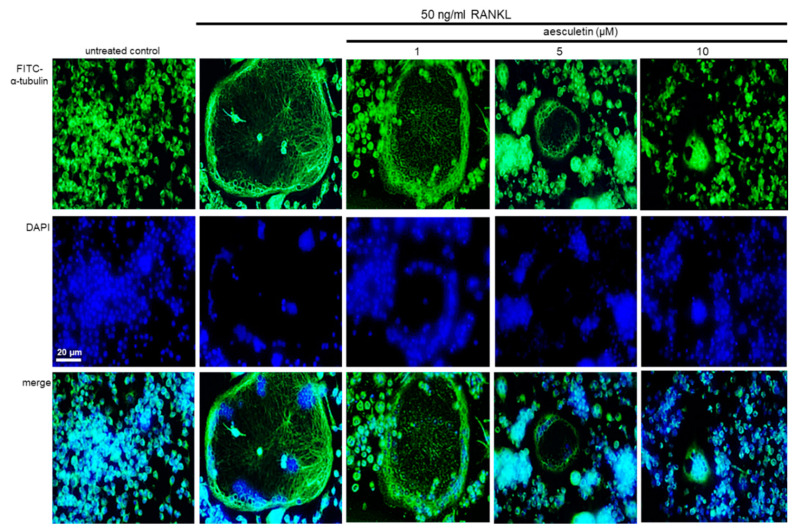
Suppressive effects of aesculetin on receptor activator of nuclear factor-κB ligand (RANKL)-induced microtubular formation in actin ring-bearing osteoclasts. Raw 264.7 macrophages were cultured in minimum essential medium alpha medium (α-MEM) with 50 ng/mL RANKL in the absence and presence of 1–10 μM aesculetin for 5 days. RANKL-differentiated cells were fixed in 4% paraformaldehyde for 10 min, and immunocytochemical analysis was conducted with a primary antibody of α-tubulin and secondary antibody of mouse immunoglobulin G (IgG) conjugated with fluorescein isothiocyanate (FITC). Subsequently, nuclear counterstaining was done with 4′,6-diamidino-2-phenylindole (DAPI). Fluorescent images were taken with a fluorescence microscope. Original magnification of microscopic images (*n* = 3). Scale bar = 20 µm.

Microtubules, one of the elements of cytoskeletons, act as a transport system that delivers vesicles and lysosomes to the ruffled border, illustrating the importance of microtubule networks in osteoclast function [20,35]. The formation of actin rings and microtubule cytoskeleton was elevated in RANKL-treated macrophages, as evidenced by immunocytochemical staining with green FITC-conjugated α-tubulin (Figure 5). When ≥5 µM aesculetin was administered to RANKL-loaded macrophages, the microtubule cytoskeleton within actin rings almost completely disappeared. Accordingly, aesculetin disturbed the microtubule cytoskeleton involved in lysosomal transport within osteoclasts, leading to osteoclastic bone resorption.

### 2.5. Blockade of Lysosome Positioning to Microtubules by Aesculetin

**Figure 6:**
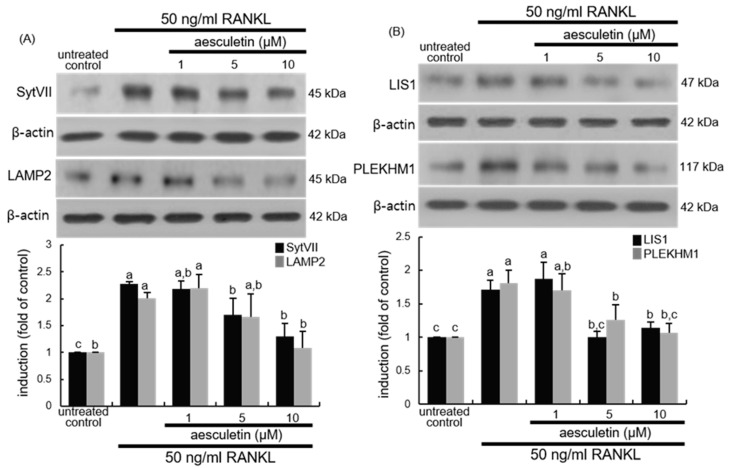
Western blot data showing inhibition of induction of synaptotagmin VII (SytVII), lysosome-associated membrane protein 2 (LAMP2), lissencephaly-1 (LIS1), and Pleckstrin homology domain-containing protein family member 1 (PLEKHM1) (**A**,**B**). Raw 264.7 cells were cultured in minimum essential medium alpha medium (α-MEM) with 50 ng/mL receptor activator of nuclear factor-κB ligand (RANKL) in the absence or presence of 1–10 μM aesculetin for 5 days. Whole-cell lysates were subject to sodium dodecyl sulphate-polyacrylamide gel electrophoresis (SDS-PAGE) and Western blot with a specific antibody against SytVII, LAMP2, LIS1, and PLEKHM1. β-Actin was used as internal control. The bar graphs (mean ± SEM, *n* = 3) represent quantitative results of blots obtained from a densitometer. Respective values in double-bar graphs not sharing a small letter are significantly different at *p* < 0.05.

Bone resorption by osteoclasts depends on vacuoles including lysosomes filled with acid phosphatase [20]. How secretory lysosomes are directed to fuse with the ruffled border is still enigmatic. Osteoclast SytVII, a lysosome-associated calcium sensor protein that regulates exocytosis, regulates transport and secretion of matrix-degrading molecules into the resorptive lacuna [21]. This study found that RANKL prompted the SytVII induction in Raw 264.7 macrophages and that such induction was attenuated in 10 μM aesculetin-supplied osteoclasts (Figure 6A). LAMP2 colocalizes with cathepsin K and Syt VII and is involved in the process of vesicular trafficking [21,36]. RANKL stimulated the LAMP2 induction in Raw 264.7 macrophages (Figure 6A). When 1–10 µM aesculetin was administered to RANKL-induced cells, the LAMP2 induction was reduced.

The process of microtubule organization and lysosomal secretion requires the interaction between microtubule regulator LIS1 and lysosome-associated PLEKHM1 [22,37]. This study found that cellular induction of LIS1 and PLEKHM1 was enhanced by an exposure of RANKL (Figure 6B). However, administering ≥ 5 μM aesculetin to RANKL-challenged macrophages inhibited the induction of LIS1 and PLEKHM1. Thus, aesculetin may lead to disorganized microtubules and abrogate the peripheral distribution of lysosomes in osteoclasts.

### 2.6. Disruption of Trafficking of Lysosomes into Ruffled Border by Aesculetin

**Figure 7:**
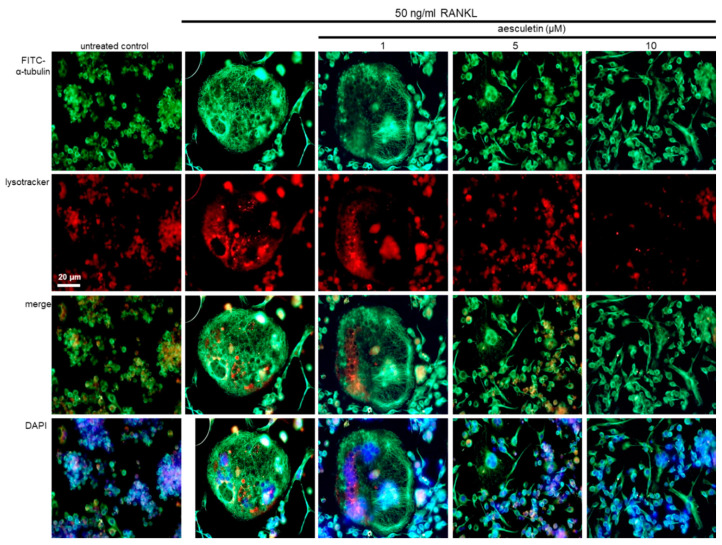
Blockade of positioning of lysosomes and microtubules in actin ring-bearing osteoclasts. Raw 264.7 cells were cultured for 5 days with 50 ng/mL receptor activator of nuclear factor-κB ligand (RANKL) in the absence or presence of 1–10 µM aesculetin. RANKL-differentiated cells were fixed in 4% paraformaldehyde for 10 min, followed by immunocytochemical analysis with a primary antibody of α-tubulin and anti-mouse IgG conjugated with fluorescein isothiocyanate (FITC). Subsequently, commercially available red fluorescent lysoTracker Red DND-99 dye was employed for the staining of lysosomes. Finally, cells were counterstained with 4′,6-diamidino-2-phenylindole (DAPI) for nuclei. Representative fluorescent images were obtained with a fluorescence microscope (*n* = 3). Scale bar = 20 µm.

This study conducted a double-staining with green FITC-conjugated anti-α-tubulin and red lysoTracker dye to detect colocalization of microtubules and lysosomes. RANKL created multinucleated giant cells with a solid microtubule cytoskeleton and heavy reddish LysoTracker fluorescence (Figure 7). However, the red staining with lysoTracker was reduced in RANKL-exposed and 10 µM aesculetin-treated cells, indicating a decrease in lysosomal flux of osteoclasts with mature cytoskeleton.

**Figure 8:**
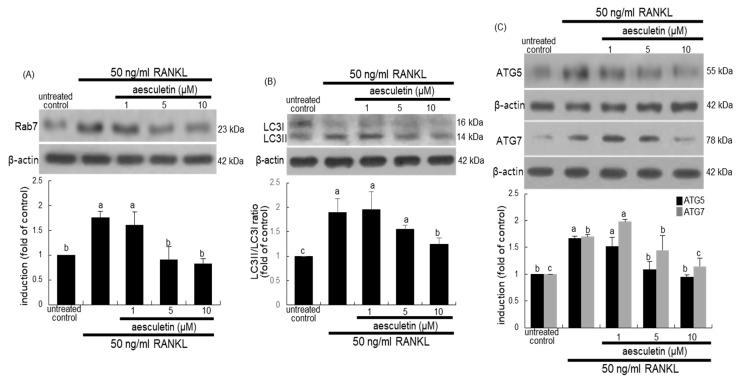
Western blot data showing inhibition of induction of Rab7 (**A**), microtubule-associated protein light chain 3 (LC3) (**B**), Atg5, and Atg7 (**C**) by aesculetin. Raw 264.7 cells were cultured for 5 days with 50 ng/mL RANKL in the absence or presence of 1–10 µM aesculetin. Whole-cell lysates were subject to SDS-PAGE and Western blot with a primary antibody against Rab7, LC3, Atg5, and Atg7. β-Actin was used as an internal control. The bar graphs (mean ± SEM, *n* = 3) represent quantitative results of blots obtained from a densitometer. Respective values in double-bar graphs not sharing a letter are significantly different at *p* < 0.05.

In osteoclasts, the formation of the ruffled border involves vesicular trafficking pathways that are regulated by the Rab family of GTPase, in particular lysosomal Rab7 [34]. The Rab7 induction by RANKL was diminished in ≥5 µM aesculetin-exposed mature osteoclasts (Figure 8A). The conjugation and lipidation of LC3 protein are responsible for fusion of secretory lysosomes into bone-apposed plasma membrane in order to form dense folds of the ruffled border [24]. The conversion of soluble LC3I to lipid-bound LC3II was enhanced in osteoclasts differentiated by RANKL (Figure 8B). In contrast, the LC3II induction was reduced by administering 10 µM aesculetin. The LC3II localization onto the ruffled border involves aegis of the Atg12–Atg5 conjugate and Atg7 [24]. RANKL promoted the induction of Atg5 and Atg7, which was blunted by adding 10 µM aesculetin during osteoclast differentiation (Figure 8C). Thus, aesculetin may block the formation of the putative ruffled border by impairing lysosomal trafficking and protease exocytosis.

## 3. Materials and Methods

### 3.1. Materials

Minimum essential medium alpha medium (α-MEM), Dulbecco’s modified eagle’s media (DMEM), aesculetin, and aesculin were purchased from Sigma-Aldrich Chemicals (St. Louis, MO, USA), as were all other reagents, unless otherwise specified. Fetal bovine serum (FBS) and penicillin–streptomycin were obtained from BioWhittaker (San Diego, CA, USA). 3-(4,5-Dimetylthiazol-yl)-diphenyl tetrazolium bromide (MTT) was supplied from DUCHEFA Biochemie (Haarlem, Netherlands). RANKL was obtained from PeproTech (Rocky Hill, NJ, USA). Antibodies of mouse V-ATPase (cat. no. sc-69108), mouse cathepsin K (cat. no. sc-48353), mouse LIS1 (cat. no. sc-374586), mouse integrin αV (cat. no. sc-6617-R), mouse Cdc42 (cat. no. sc-8401), and mouse α-tubulin (cat. no. sc-8035) were obtained from Santa Cruz Biotechnology (Dallas, TX, USA). Antibodies of mouse CAII (cat. no. MAB2184) and mouse MMP-9 (cat. no. MAB936) were provided by R&D Systems (Minneapolis, MN, USA). Antibodies of mouse Rab7 (cat. no. ab137029), mouse ClC-7 (cat. no. ab86196), and mouse Syt VII (cat. no. ab174633) were provided by Abcam (Cambridge, UK). Antibodies of mouse Atg7 (cat. no. 4445), mouse Atg5 (cat. no. 4445), mouse CD44 (cat. no. 37259), and mouse integrin β3 (cat. no. 4702) were supplied by Cell Signaling Technology (Beverly, MA, USA). Human LC3 antibody (cat. no. M152-3) was obtained from MBL International (Woburn, MA), and mouse Ae2 (cat. no. NBP1-59858) antibody was purchased from Novus Biologicals (Littleton, CO, USA). Antibodies of mouse PLEKHM1 (cat. no. bs-8062R) and LAMP2 (cat. no. bs-2379R) were provided by Bioss Antibodies (Woburn, MA, USA). Horseradish peroxidase (HRP)-conjugated goat anti-rabbit, goat anti-mouse, and donkey anti-goat immunoglobulin G (IgG) were provided by Jackson Immuno Research Laboratories (West Grove, PA, USA).

Aesculetin and aesculin were dissolved in dimethyl sulfoxide (DMSO) for live culture with cells; the final culture concentration of DMSO was < 0.5%.

### 3.2. Osteoclast Differentiation of Raw 264.7 Cells

Murine macrophage Raw 264.7 cells (American Type Culture Collection, Manassas, VA, USA) were cultured in DMEM containing 10% FBS, 2 mM glutamine, 100 U/mL penicillin, and 100 µg/mL streptomycin at 37 °C in a humidified atmosphere of 5% CO_2_ in air. For osteoclast differentiation, Raw 264.7 cells were plated on 24-well plates at the density of 1 × 10^4^ cells/mL and cultured for 5 days in α-MEM containing 10% FBS and 50 ng/mL RANKL in the absence and presence of 1–15 µM aesculetin or 1–40 µM aesculin. Cell culture medium was newly changed every 2 days.

The cytotoxicity of aesculetin and aesculin was determined using an MTT assay. Cells were treated with 1 mg/mL MTT solution and incubated at 37 °C for 3 h, resulting in the formation of an insoluble purple formazan product that was dissolved in 250 µL of isopropanol. Optical density was measured using a microplate reader at λ = 570 nm. Raw 264.7 cell viability was not influenced by administering ≤ 10 µM aesculetin and ≤ 40 µM aesculin in the absence (Figure 1B and Figure 2B) and presence of 50 ng/mL RANKL (Figure 1C and Figure 2C).

### 3.3. Measurement of Tartrate-Resistant Acid Phosphatase (TRAP) Activity

Cells were fixed with 4% formaldehyde for 10 min and then were incubated in 50 mM citrate buffer (50 mM citric acid and 50 mM sodium citrate (pH 4.5)) containing 5 mM 4-nitrophenylphosphate, and 10 mM sodium tartrate for 1 h. The reaction was terminated by adding 0.1 N NaOH. Absorption intensity was measured using a microplate reader at λ = 405 nm. Furthermore, cells were fixed with 4% formaldehyde and stained for 30 min with a commercially available TRAP kit (Sigma-Aldrich Chemical, St. Louis, MO, USA). TRAP-positive multinucleated osteoclasts were visualized under light microscopy (ECLIPSE Ni-U, Nikon, Tokyo, Japan).

### 3.4. Western Blot Analysis

Cell lysates were prepared from Raw 264.7 cells differentiated with RANKL. Equal amounts of lysate proteins or equal volumes of culture media were electrophoresed on 6–20% sodium dodecyl sulphate-polyacrylamide gel electrophoresis (SDS-PAGE) gels and transferred onto a nitrocellulose membrane. Nonspecific binding was blocked by soaking membranes in a Tris-buffered saline/Tween-20 (TBS-T) buffer (50 mM Tris-HCl (pH 7.5), 150 mM NaCl, and 0.1% Tween-20) containing 3% bovine serum albumin or 5% nonfat milk for 3 h. The membranes were incubated with a primary antibody against CAII, V-ATPase, ClC-7, cathepsin K, Rab7, Atg5, Atg7, Syt VII, Ae2, CD44, integrin αV, integrin β3, MMP-9, LC3, PLEKHM1, LAMP2, Cdc42, or LIS1. The membranes were then incubated with goat anti-rabbit, goat anti-mouse, or donkey anti-goat IgG conjugated to HRP as a secondary antibody. The protein levels on gels were measured by using ECL chemiluminescent detection reagents (Millipore, Billerica, MA, USA) and Konica X-ray film (Konica, Tokyo, Japan). Incubation with β-actin antibody was conducted for comparative control.

The bone resorption assay was performed using a resorption assay kit (CSR-BRA-48X2KIT, Cosmo Bio Co., Tokyo, Japan). Raw 264.7 cells were seeded on calcium phosphate-coated well plates at a density of 1 × 10^4^ cells/mL and cultured for 5 days in phenol red-free α-MEM containing 10% FBS and 50 ng/mL RANKL in the absence and presence of 1–10 µM aesculetin. To measure resorbed pit areas, the cells were washed in 5% NaOCl. The resorption pits developed on the plate were visualized under light microscopy (ECLIPSE TS100, Nikon, Tokyo, Japan).

### 3.5. Actin Ring Staining

The formation of actin rings was determined by staining actin filaments with rhodamine phalloidin (Life Technologies, Carlsbad, CA, USA). RANKL-differentiated Raw 264.7 cells were fixed with 4% formaldehyde for 10 min and washed with prewarmed phosphate-buffered saline (PBS). Cells were incubated for 30 min with 10 units of rhodamine phalloidin. Fluorescent images were taken with a fluorescence microscope (Carl Zeiss, Oberkochen, Germany).

### 3.6. Immunochemical Staining

Raw 264.7 cells grown on glass coverslips were differentiated for 5 days in α-MEM containing 10% FBS and 50 ng/mL RANKL in the absence and presence of 1–10 µM aesculetin. After permeabilizing on ice with 0.1% Triton X-100 and 0.1% sodium citrate for 1 min, α-tubulin as a primary antibody was added and incubated overnight at 4 °C. After several washes with PBS/Tween-20, cells were incubated with anti-mouse IgG conjugated with a green-fluorescent dye fluorescein isothiocyanate (FITC). Following washes, nuclear counterstaining was conducted using 4′,6-diamidino-2-phenylindole (DAPI) for 15 min.

Another experiment was performed for the detection of acidic lysosomes. Cells were incubated for 1 h with 50 nM acidotropic probe LysoTracker Red DND-99 (Life Technologies, Carlsbad, CA, USA). Subsequently, cells were fixed with 4% ice-cold formaldehyde and blocked nonspecific binding with 20% FBS in PBS for 1 h. Immunocytochemical analysis was further conducted for the staining of FITC-conjugated α-tubulin. Each slide was mounted in a mounting medium and images of each slide were taken using an optical fluorescence microscope (Zeiss, Oberkochen, Germany).

### 3.7. Statistical Analysis

The results were presented as means ± SEM for each treatment group. Statistical analyses were performed using Statistical Analysis Systems statistical software package (SAS Institute Inc., Cary, NC, USA). Significance was determined by one-way ANOVA, followed by Duncan’s range test for multiple comparisons. Differences were considered significant at *p* < 0.05.

## 4. Discussion

**Figure 9:**
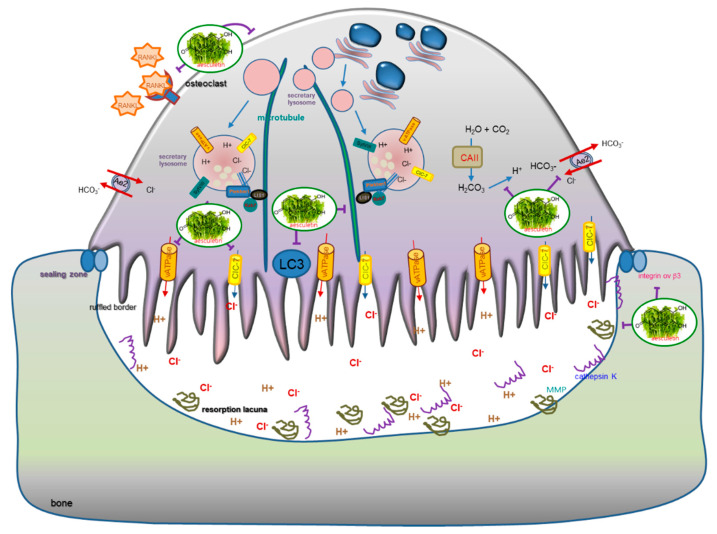
Schematic diagram showing the inhibitory effects of aesculetin on ruffled border formation and lysosomal trafficking responsible for bone resorption. As depicted, aesculetin blocks the RANKL signaling process responsible for bone resorption. The symbol I^−^ indicates sites of inhibition manifested by aesculetin.

Seven major findings were extracted from this study. (1) Nontoxic aesculetin but not aesculin at ≤10 μM showed a marked inhibition of the formation of TRAP-positive osteoclasts. (2) RANKL promoted the induction of CAII, Ae2, ClC-7, and V-ATPase in Raw 264.7 macrophages, which was deterred by aesculetin. (3) The presence of aesculetin attenuated the cathepsin K expression and MMP-9 secretion of osteoclasts in parallel with its reduction of the resorption pits and the actin ring formation up-regulated by RANKL. (4) When Raw 264.7 cells were treated with 50 ng/mL RANKL, aesculetin was effective in reducing the induction of αv integrin and β3 integrin but not core-linking CD44. (5) The induction of Cdc42, Atg5, Atg7, SytVII, and LAMP-2 was diminished by administering 10 μM aesculetin during osteoclast differentiation, indicating that aesculetin may suppress formation of lysosomes and ruffled border crucial for bone resorption. (6) Aesculetin inhibited the induction of LIS1 and PLEKHM1 in RANKL-exposed osteoclasts, suggesting that aesculetin blocked lysosome positioning to microtubules involved in the lysosomal transport within osteoclasts. (7) The induction of LC3II and Rab7 by RANKL was diminished in ≥5 µM aesculetin-exposed mature osteoclasts. Thus, aesculetin may block the formation of the putative ruffled border through impairing lysosomal trafficking and protease exocytosis (Figure 9).

Osteoclasts are giant multinucleated hematopoietic cells specialized for bone resorption [7,8]. Bone remodeling comprises finely coordinated steps of activation, resorption, reversal, formation, and termination via activation of tightly coupled signaling of RANK/RANKL/OPG [4,5,9]. OPG is secreted by osteoblasts and osteogenic stromal stem cells and protects the skeleton from excessive bone resorption through binding to RANKL and hampering interaction with RANK [38]. On the other hand, the remodeling is regulated by signaling of various calcitropic hormones, proinflammatory cytokines, and growth factors [1,5,39]. Signaling of transforming growth factor-β and parathyroid hormone is known to control bone remodeling and maintenance [40]. In addition, the Wnt signaling pathway manipulates bone formation and remodeling [41,42]. Mechanistic signaling pathways involved in bone remodeling can be targets for pharmacological interventions for antiresorptive and bone-forming therapies [10,11,12]. In particular, the main classes of antiresorptives currently in use are calcium, bisphosphonates, estrogen, selective estrogen receptor modulators, and calcitonin [43]. However, the mechanistic efficacy and safety of such therapeutic interventions remain to be confirmed.

The ruffled border of osteoclasts in contact with the bone surface is formed by massive trafficking of secretory lysosomes [14,15]. Bone resorption by osteoclasts highly depends on exocytosis of lysosomes full of proteases, acid hydrolases, and acid phosphatases [15]. Considerable efforts have been made in seeking naturally occurring compounds as novel therapeutic agents targeting against bone loss [25,26,44]. One study shows that aesculetin inhibits osteoclast differentiation through suppressing expression of c-Fos and nuclear factor of activated T cell c1 [45]. This study found that aesculetin abrogated the intracellular induction of TRAP, cathepsin K, and MMP-9 from RANKL-loaded macrophages. Osteoclastic activity resorbing the mineralized bone matrix entails acidification of resorption lacuna generated by contact of osteoclasts with the bone surface [15,16]. Previous studies showed that phloretin and fisetin blunted bone resorption through inhibition of acidification of resorption lacuna of TRAP-positive osteoclasts [46,47]. Nontoxic aesculetin but not aesculin inhibited RANKL induction of CAII, Ae2, ClC-7, and V-ATPase necessary for the extracellular acidification in osteoclasts. It should be noted that the glucose moiety of aesculin interfered with the inhibitory effects on RANKL-induced osteoclast differentiation. Accordingly, the defect of bone-resorbing proteases and proton providers by aesculetin failed to digest organic components of bone matrices. Furthermore, the bone-resorbing activity of osteoclasts counts on a tight integrin-based adhesion to the bone surface, forming an actin-rich sealing zone [48]. During the bone resorption of RANKL-loaded macrophages, aesculetin suppressed the formation of podosome belts (actin rings), through deterring the induction of αv integrin and β3 integrin but not podosome core-linking CD44. Small GTPases of Rac and Cdc42 are crucial for actin polymerization, podosome organization, and the assembly of the sealing zone in coordination with integrin signaling [34]. Aesculetin retarded the Cdc42 induction in RANKL-loaded macrophages. Osteoclasts generated from Cdc42-knockout mice reduce actin ring formation and bone resorption [49]. It should be noted that aesculetin inhibited the formation of the microtubule cytoskeleton within the actin ring but not the organization of the microtubule architecture.

Emerging evidence suggests that several membrane trafficking pathways including endocytic, secretory, transcytotic, and autophagic pathways are involved in operating the subdomains of ruffled border in osteoclasts [13,15]. The means by which secretory lysosomes directly fuse with the ruffled border are elusive. The autophagic proteins of the Atg12–Atg5 conjugate, Atg7, Atg4B, and LC3 are involved in fusion of secretory lysosomes with the ruffled border, promoting bone resorption activity [23,24,50]. Atg5-dependent LC3II localization onto the osteoclast ruffled border promotes fusion with secretory lysosomes [24]. The deletion of Atg5 or Atg7 suppresses trafficking of cathepsin K-containing lysosomes at the ruffled border and inhibits secretion of lysosomal contents into the resorption lacuna [20,24]. This study found that aesculetin inhibited the induction of LC3, Atg5, and Atg7 involved in LC3 lipidation of osteoclasts, indicating that this compound inhibited fusion of LC3II-decorated membrane with secretory lysosomes. Similarly, gossypetin diminished the ATG induction and lysosomal cathepsin K activity in actin ring-bearing osteoclasts [27]. Furthermore, it was shown that LC3 associates with microtubules in proximity of the actin ring and regulates Cdc42 [23]. The inhibition of actin ring formation by aesculetin might be ascribed to failure of the regulatory link among the LC3, microtubules, and Cdc42 [23]. In addition, another small GTPase Rab7 is crucial for vesicular trafficking to the ruffled border in an Atg5-dependent manner [24]. The Rab7 induction by RANKL was diminished in aesculetin-bearing mature osteoclasts. It can be assumed that aesculetin impaired fusion of secretory lysosomes with bone-apposed plasma membrane, leading to decreased bone resorption of osteoclasts.

There is recent evidence demonstrating that molecular machinery governs the positioning and peripheral distribution of lysosomes in osteoclasts [20,37]. Distinct vesicular transport in osteoclasts may be exploited for the development of pharmacological therapies for bone disorders [13]. The lysosomal exocytosis is mediated by SytVII colocalized with LAMP2 and cathepsin K on lysosomes and promotes the fusion of lysosomes with the ruffled border [21,51]. In this study, aesculetin inhibited cellular induction of SytVII and LAMP2 by the presence of RANKL, indicating defect of formation and exocytosis of secretary vesicles during bone resorption. Furthermore, loss of connection of lysosomes to microtubules impairs lysosomal distribution in osteoclasts leading to failure of bone resorption [20]. LIS1 regulates osteoclastic function through interactions with PLEKHM1 and dynein moving along microtubules [22]. Moreover, the Rab7-associated molecular machinery with LIS1 and PLEKHM1 mediates lysosome peripheral positioning in osteoclasts [22,37]. This study showed that aesculetin hampered the interaction between microtubules and lysosomes by virtue of its ability to inhibit the induction of Rab7, LIS1, and PLEKHM1. These results suggest that Rab7/LIS1/PLEKHM1-mediated positioning to microtubules and movement of lysosomes along with microtubules may be a new therapeutic target for bone diseases with increased resorption. Since this study focused on exploring lysosomal mechanisms involved in anti-resorptive effects of aesculetin, the potential antiosteoclastogenetic mechanism(s) of aesculetin should be elucidated.

In summary, the current study demonstrated that aesculetin abrogated the RANK signaling-triggered multinucleated osteoclast formation and bone resorption in macrophages. Aesculetin hampered the actin ring formation along with deterring cellular induction of αvβ3 integrin and Cdc42. This compound failed to create the acidic resorption lacuna at the osteoclast–bone matrix interface through dampening the induction of proton pump and chloride channel on the ruffled border and anion exchanger on the contra-lacunar membrane domain. Furthermore, aesculetin inhibited the fusion of lysosomes with the ruffled border through disturbing induction of Atg and LC3 proteins and blunted SytVII- and LAMP2-mediated lysosomal exocytosis. Finally, aesculetin deranged lysosome positioning to microtubules and lysosomal movement mediated by the Rab7/LIS1/PLEKHM1-associated trafficking machinery. Although aesculetin may serve as a modulator against osteoclastogenesis under in vitro conditions, its dietary role as an osteoprotective agent remains elusive.

## Abbreviations

Ae2	anion exchange protein 2
Atg	autophagy-related proteins
CAII	carbonic anhydrase II
ClC-7	chloride channel 7
LAMP2	lysosome-associated membrane protein 2
LC3	light chain 3
LIS1	lissencephaly-1
OPG	osteoprotegerin
PLEKHM1	Pleckstrin homology domain-containing protein family member 1
MMP	matrix metalloproteinase
RANK	receptor activator of nuclear factor-κB
RANKL	RANK ligand
Syt VII	synaptotagmin VII
TRAP	tartrate-resistance acid phosphatase
V-ATPase	vacuolar-type H(+)-ATPase
